# Anticancer Effects of I-BET151, an Inhibitor of Bromodomain and Extra-Terminal Domain Proteins

**DOI:** 10.3389/fonc.2021.716830

**Published:** 2021-09-02

**Authors:** Jiacheng Lai, Ziqiang Liu, Yulei Zhao, Chengyuan Ma, Haiyan Huang

**Affiliations:** Department of Neurosurgery, The First Hospital of Jilin University, Changchun, China

**Keywords:** cancer, bromodomain and extra-terminal domain protein, I-BET151, signal transduction, drug combination

## Abstract

I-BET151 is an inhibitor of bromodomain and extra-terminal domain (BET) proteins that selectively inhibits BET family members (BRD2, BRD3, BRD4, and BRDT). Over the past ten years, many studies have demonstrated the potential of I-BET151 in cancer treatment. Specifically, I-BET151 causes cell cycle arrest and inhibits tumor cell proliferation in some hematological malignancies and solid tumors, such as breast cancer, glioma, melanoma, neuroblastoma, and ovarian cancer. The anticancer activity of I-BET151 is related to its effects on NF-κB, Notch, and Hedgehog signal transduction pathway, tumor microenvironment (TME) and telomere elongation. Remarkably, the combination of I-BET151 with select anticancer drugs can partially alleviate the occurrence of drug resistance in chemotherapy. Especially, the combination of forskolin, ISX9, CHIR99021, I-BET151 and DAPT allows GBM cells to be reprogrammed into neurons, and this process does not experience an intermediate pluripotent state. The research on the anticancer mechanism of I-BET151 will lead to new treatment strategies for clinical cancer.

## Introduction

Bromodomain and extra-terminal domain (BET) proteins function as epigenetic readers that mainly recognize acetylated lysine residues in chromatin proteins. The BET family consists of four members, among which BRD2, BRD3, and BRD4 are ubiquitously expressed, and BRDT is only expressed in the testis. Conserved structural components of these proteins include two characteristic bromine domains (BD1 and BD2) and an extra-terminal domain (ET), along with a C-terminal domain (CTD) found only in BRD4 and BRDT (1). BET proteins participate in the formation of multiple nuclear protein complexes and play an important role in regulating gene transcription, as well as DNA replication, damage, and repair (2).

The abnormal manifestations of BET family members, especially BRD2 and BRD4, occur in various cancer types. In nuclear protein in testis (NUT) midline carcinoma (NMC), BRD3 and BRD4 fuse with NUT and retain it in the nucleus, which interferes with the differentiation of epithelial cells and promotes cancer growth (3). In melanoma, glioma, ovarian cancer, and some other cancers, the overexpression of BRD2 and BRD4 is associated with poor prognosis, and their presence affects the pathways of nuclear factor-κB (NF-κB), Notch, and Hedgehog (Hh) signaling (4–6). The changes in the expression and distribution of BET family members in different cancer cells and even stem cells often promote the occurrence and development of cancer.

**Figure 1:**
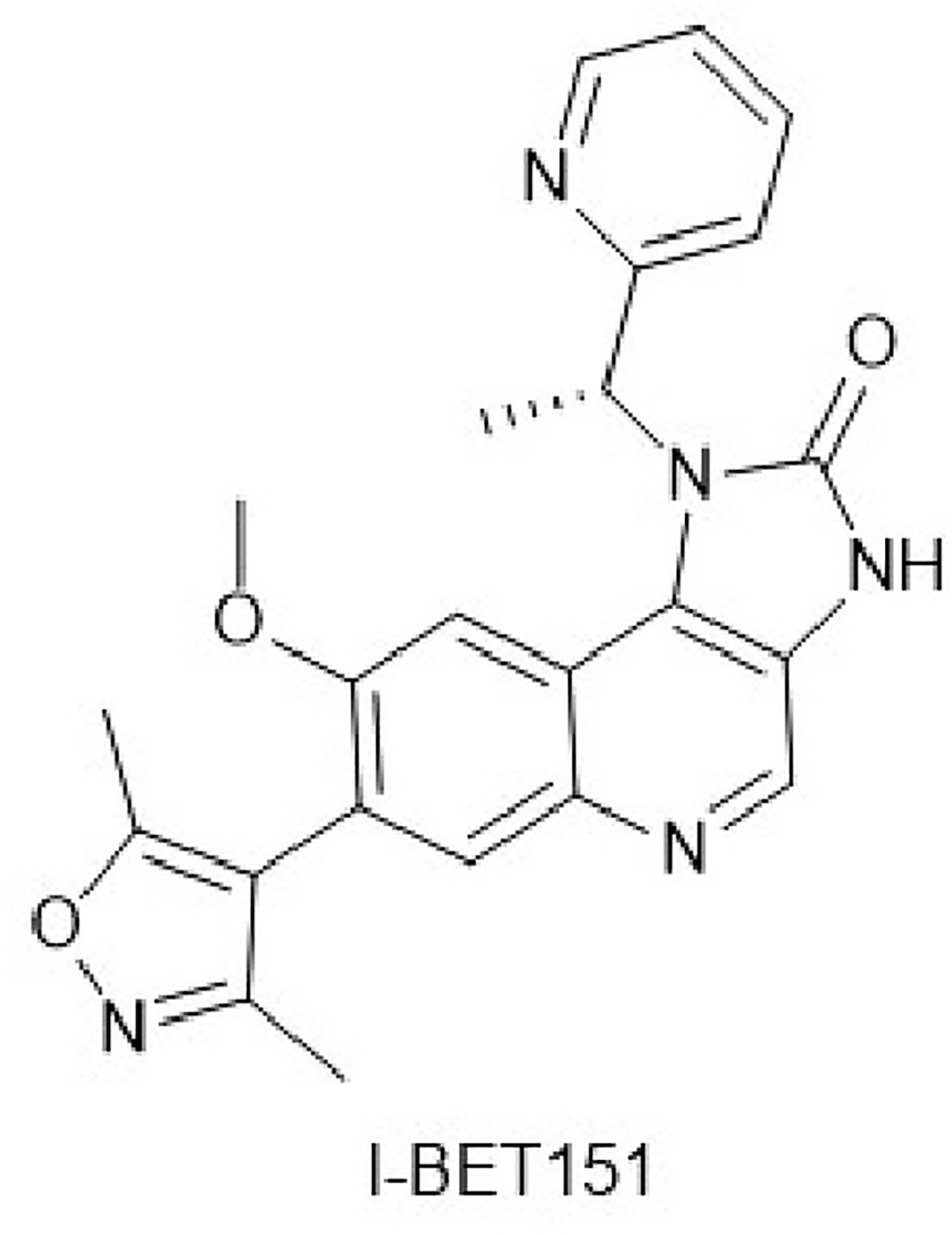
Chemical structure of I-BET151.

I-BET151 (**Figure 1**) is a new type of BET protein inhibitor with the chemical designation 7-(3,5-dimethyl-4-isoxazo1lyl)-8-(methyloxy)-1-[(1R)-1-(2-pyridinyl)ethyl]-1,3-dihydro-2H-imidazo[4,5-c]quinolin-2-one, and the molecular formula C_23_H_21_N_5_O_3_. In 2011, Dawson et al. developed and optimized I-BET151 as a BET inhibitor with good bioavailability and a prolonged terminal half-life. I-BET151 binds into BD1 acetyl-lysine recognition pocket and displaces BET proteins from nuclear chromatin. Of the 27 bromodomain proteins in the HL60 nuclear extract, the excess I-BET151 affects only BRD2, BRD3, BRD4, and BRD9. Among them, the effect of I-BET151 on BRD9 may be indirect because BRD9 and BRD4 form a complex.I-BET151 selectively inhibits leukemia mouse models and mixed-lineage leukemia (MLL) primary patient samples, and its half-life is significantly longer than that of similar BET inhibitors (JQ1, I-BET762) (7). Several recent studies have demonstrated the anticancer effects of I-BET151 on various solid tumors, apart from leukemia, which has attracted extensive attention (4–6). In this review, we will discuss the existing research on anticancer effects of I-BET151 and focus on the implications for cancer therapy.

## The Anticancer Mechanism of I-BET151

**Figure 2:**
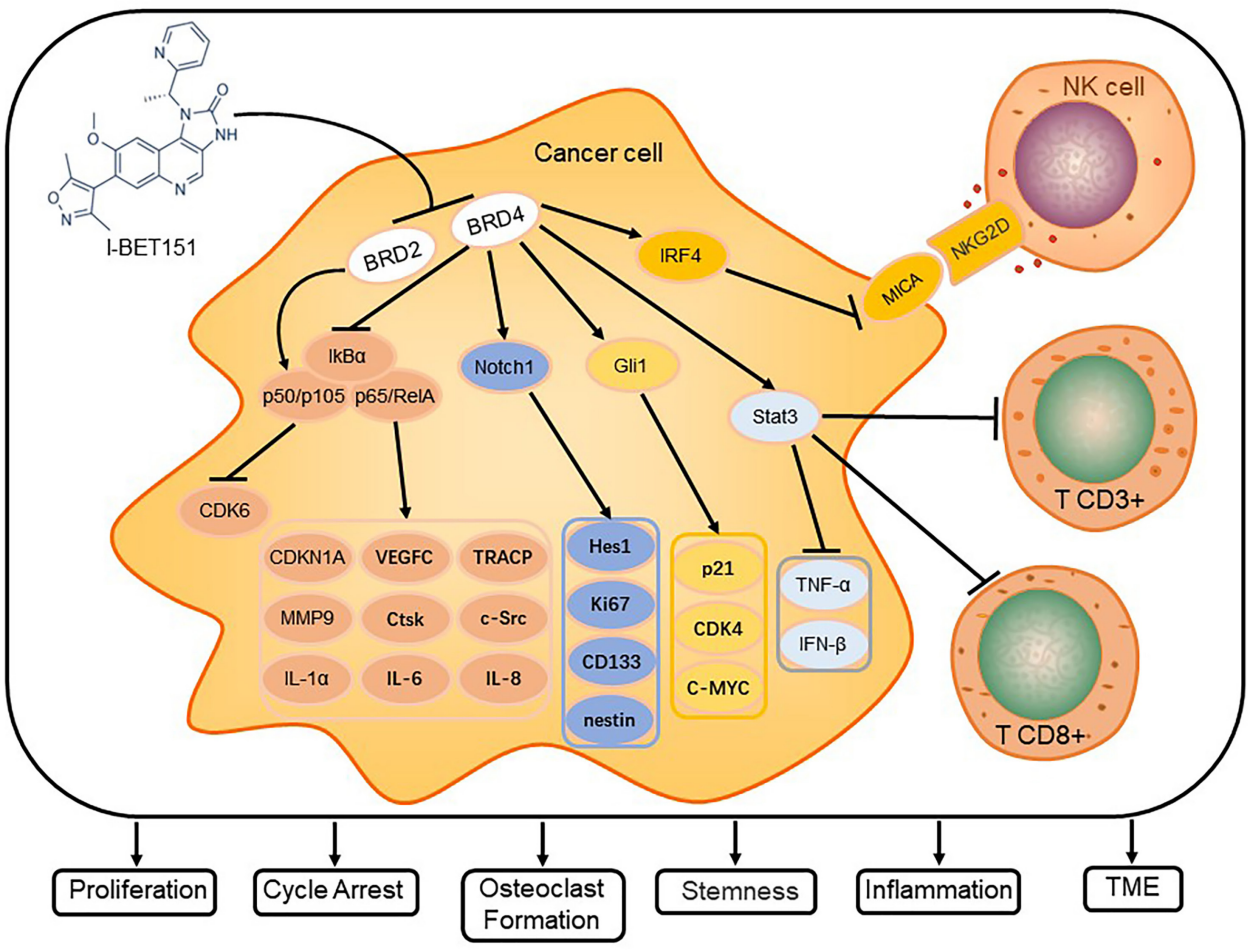
I-BET151 affects the signal transduction in cancer cells and modulates critical cellular processes. I-BET151 specifically inhibits BRD2 and BRD4, decreases the intracellular content of p50/p105, diminishes the degradation of IkB-α, prevents the dissociation of p50/p105 and p65/RelA from IkB-α and their transport into the nucleus, and decreases the activity of NF-B signal transduction. In addition, I-BET151 reduces the binding of BRD4 to the Notch1 and Gli1 promoter regions, inhibits the transcription of Notch1 and Gli1, and causes the target molecules of Notch and Hh signaling pathways to change. In the aspect of influencing tumor microenvironment, I-BET151 not only targets BRD4, which leads to the increase of MICA expression and promoting NK cell degranulation, but also inhibits Stat3 signal, which leads to more CD3+ and CD8+ cells in tumors. Eventually, I-BET151 leads to cell cycle arrest, inhibition of cancer cell proliferation, stemness of stem cells, inflammatory factor release, osteoclast formation and regulation of TME.

I-BET151 selectively inhibits members of the BET family, which affects intracellular signal transduction pathways, tumor microenvironment (TME), and telomere length mainly *via* the pathways for NF-κB, Notch, and Hh signaling (**Figure 2**).

### I-BET151 and Inhibition of NF-κB Signaling Pathways

As early as 1863, Rudolf Virchow linked inflammation with cancer. Subsequent studies suggested that inflammation may be an auxiliary factor in cancer (8). According to the global cancer attribution analysis in 2018, 2.2 million cancer cases were attributed to infections, and inflammation was the main component of these chronic infections (9). The seven members of the NF-κB family, RelA/p65, c-Rel, RelB, p100, p52, p105, and p50, are central mediators of inflammatory processes. Moreover, there is growing evidence that the NF-κB signaling pathway forms a critical connection between inflammation and cancer. Specifically, NF-κB can stimulate cancer cell proliferation, inhibit cancer cell apoptosis, and promote cancer-related migration and invasion in various cancers (10).

I-BET151 treatment reduces NF-κB activity in many melanoma cell lines, especially SK-Mel-28 and Mel-JD, and in primary cell lines with vemurafenib resistance, which is related to NF-κB overexpression. NF-κB activity inhibition by I-BET151 is mainly reflected in the reduction of p105 and p50, whereas RelA remains unchanged, which has also been confirmed in tumor-bearing animal models. Moreover, the expression of CDKN1A is increased while the CDK6 content is decreased, which indicates that I-BET151 is reducing cancer cell proliferation, resulting in cell cycle arrest. Furthermore, in melanoma, I-BET151 also inhibits the production of cytokines and chemokines, such as interleukin (IL)-1α, vascular endothelial growth factor C (VEGFC), IL-6, and IL-8, and its effect on NF-κB is mainly mediated *via* BRD2 (4).

Monocytes of patients with myeloma easily differentiate into osteoclasts because NF-κB signaling is activated in monocytes by the receptor agonist of NF-κB ligand (RANKL), leading to IκB-α degradation and RelA/P65 nuclear translocation, both of which promote osteoclast generation. I-BET151 specifically inhibits BRD4, thereby inhibiting RANKL-induced IκB-α degradation and p65 nuclear translocation. In isolated mononuclear cells from healthy donors and patients with multiple myeloma, I-BET151 inhibits NF-κB signaling pathways in monocytes in a dose-dependent manner and diminishes the expression of osteoclast-specific genes, such as *TRACP*, *MMP9*, *Ctsk*, and *c-Src*, all of which contributes to the inhibition of osteoclast formation. Moreover, BRD4 knockdown also enhances the effect of I-BET151 (11).

Thus, I-BET51 inhibits NF-κB signal by targeting different molecules (BRD2 or BRD4), which is caused by different cell types. However, abnormally activated NF-κB signaling may induce I-BET151 resistance in tumors, as demonstrated by triple-negative breast cancer (TNBC) and lymphoma cell line U937. In these and other similar cases, a select combination with other NF-κB pathway inhibitors can restore the susceptibility of tumor cells to I-BET151 (12, 13).

### I-BET151 and Inhibition of Notch Signaling Pathways

The evolutionarily conserved Notch signaling pathway regulates cell fate during the development and the maintenance of tissue steady state; it also affects cell differentiation, proliferation, apoptosis, and epithelial-mesenchymal transition (EMT), as well as self-renewal and differentiation of stem cells (14–16). Notch signaling is related to both carcinogenesis and cancer suppression, depending on the context. In most studies, Notch appeared to be carcinogenic. However, some reports also indicate that the attenuation of Notch activity can induce certain types of brain cancer, breast cancer, ovarian cancer, small cell lung cancer, and hematologic malignancies (15, 17).

In the Notch signaling cascade, both the signal-inducing and -receiving cells interact with each other *via* ligand-receptor interactions. Mammals have four Notch receptors (Notch1-4) and five Delta-Serrate-Lag ligands (JAG1, JAG2, DLL1, DLL3, and DLL4) (18). Notch1 is activated *via* ligand-mediated cleavage by members of a disintegrin and metalloproteinase (ADAM) family and the γ-secretase complex (19). It promotes tumorigenesis in various tumor types and interferes with several signaling pathways, affecting cell proliferation, apoptosis, chemotherapy sensitivity, immune response, and self-renewal of cancer stem cells (20).

BRD4 binds to the proximal region of the TNBC Jagged1 promoter and affects migration and invasion of TNBC by regulating the Jagged1/Notch1 signaling pathway (21). Chromatin immunoprecipitation (ChIP) experiments demonstrated that BRD4 also has an affinity for the Notch1 promoter region. The inhibition and consumption of BRD4 downregulated Notch1 and suppressed stem cell marker‐related genes in glioma-initiating cells (GICs), which affected the self-renewal ability and tumorigenesis of these cells. Moreover, inhibiting Notch1 in BRD4 overexpressing cells, the self-renewal ability and proliferation of GICs are still inhibited. I-BET151 disrupted the effect of BRD4 on the Notch1 promoter by competing for acetylated histone binding sites. An immunohistochemistry analysis of intracranial orthotopic xenografts in female nude mice also found that the I-BET151 treatment suppressed the expression of Notch1, Hes1, Ki67, CD133, and nestin (6).

### I-BET151 and Inhibition of Hh Signaling Pathways

The Hh pathway is evolutionarily conserved and necessary for normal embryo development. Specifically, the Hh gene family is involved in controlling the left-right asymmetry, the polarity of the central nervous system (CNS), body segments and limbs, organogenesis, chondrogenesis, and spermatogenesis (22, 23). A recent study found that abnormal Hh signal transduction can induce various cancers, including medulloblastoma, basal cell carcinoma, rhabdomyosarcoma, breast cancer, lung cancer, liver cancer, pancreatic cancer, gastric cancer, colon cancer, and prostate cancer (24).

In mammals, the core components of the Hh pathway include three Hh ligands (Sonic hedgehog, Indian hedgehog, and Desert hedgehog), the transmembrane receptor Patched (PTCH1), the G protein-coupled receptor-like transmembrane protein Smoothened (SMO), and three transcription factors (GLI1, GLI2, and GLI3). Moreover, the primary cilia are also involved in the signal transduction of the Hh pathway (24).

I-BET151 dose-dependently attenuates Hh signal transduction in Light2 cells, and its mechanism does not depend on inhibiting the SMO activity by binding, which is employed by most Hh inhibitors. An analysis of the expression levels of PTCH1, SMO, GLI2, and GLI1 after the treatment with I-BET151 demonstrates that the inhibitor significantly reduces the expression of GLI1 but has no effect on the SMO expression level. Furthermore, I-BET151 treatment reduces the expression levels of p21, CDK4, and C-MYC. In addition, the downregulation of BRD4 with siRNA also diminishes the GLI1 expression level. This indicates that I-BET151 inhibits GLI1 transcription by limiting the binding of BRD4 to the proximal regulatory region of the GLI1 locus. In a mouse model of Ptch1+/- derived medulloblastoma, I-BET151 treatment dose-dependently reduces the viability of isolated cancer stem cells, significantly suppresses the growth of medulloblastoma *in vivo*, and lowers the expression level of the Hh target gene GLI1 (25).

### I-BET151 and Regulation of TME

TME is the cellular environment in which tumor cells are located, which is composed of a variety of cellular and non-cellular elements. Cells that TME involves include cancer-associated fibroblasts, natural killer (NK) cells, tumor-associated macrophages, tumor-associated neutrophils, tumor endothelial cells, pericytes, tumor-associated adipocytes, B lymphocytes or T lymphocytes. Non-cellular elements include blood vessels, lymphatic vessels, extracellular matrix, soluble molecules, and small organelles. TME is involved in tumor development, invasion, metastasis, recurrence, drug response, and maintenance of stem-like phenotype (26).

NK cells are the main effector cells in innate immunity, which kill cells by secreting granzymes and perforins. It interacts with extracellular matrix, cancer cells, stromal cells, and metabolites in TME to exert antitumor immunity (27). MHC class I polypeptide-related sequence A (MICA) is a natural killer group 2D ligand (NKG2DL) expressed by tumor cells. Natural killer group 2D (NKG2D) receptors activated on the surface of NK cells can bind to MICA to activate NK cells and kill tumor cells (28). I-BET151 targets BRD4 in multiple myeloma cells and inhibits the expression of C-MYC and IRF4, thereby improving the transcription and translation levels of MICA, promoting the degranulation of NK cells and inducing anti-tumor immune response (29). Multiple myeloma cells can secrete a variety of inflammatory cytokines, which interact with TME to induce osteoclast differentiation and inhibit osteoblast formation, thus promoting the development of multiple myeloma. I-BET151 inhibits the release of IL-1β, and IL-6 in peripheral blood mononuclear cells and myeloma cells by reducing BRD4-mediated activation of NF-κB (11). Furthermore, in melanoma, I-BET151 also inhibits the production of cytokines and chemokines, such as IL-1α, VEGFC, IL-6, and IL-8. This is also attributed to the inhibition of the BET family proteins by I-BET151 (4). In the ovarian cancer mouse model, I-BET151 treatment inhibits the Stat3 signaling pathway, induces more CD3+ and CD8+ cells in the tumor, increases TNF-α and IFN-β mRNA levels in the tumor and mouse spleen, and induces an anti-tumor immune response (30).

### I-BET151 Prevents Telomere Elongation

Telomeres are composed of tandem repeats of the TTAGGG sequence motif. They are special chromatin structures that form the end of the chromosome. Over multiple rounds of cell division, telomeres gradually lose the TTAGGG tandem repeats and become shorter, which is a sign of aging in organisms. Telomere length is regulated by chromatin modification, telomere binding proteins, and telomerase (31). Importantly, the risk of cancer is increased by telomeres that are too long or too short (32).

Telomerase lengthens telomeres and keeps their length in a steady state. Most cancer cells modulate telomerase activity. Therefore, telomerase inhibitors represent a targeted strategy for cancer treatment (33). Interestingly, telomere extension induced by telomerase overexpression in 293T cells can be dose-dependently blocked by I-BET151. However, treatment of these 293T cells with the highest tolerated I-BET151 dose does not inhibit the telomerase activity, indicating that I-BET151 does not employ the same mechanism for blocking telomere elongation as conventional telomerase inhibitors. The results obtained with I-BET151 are similar to those observed with three known BRD4 inhibitors, suggesting that attenuation of telomere elongation by I-BET151 depends on the inhibition of BRD4. I-BET151 interferes with the binding of BRD4 to acetylated lysine residues by targeting the bromine domain (34). It is not completely clear how BRD4 coordinates telomere maintenance, but it is known that BRD4 selectively controls the expression of telomerase reverse transcriptase in the presence of cancer-related promoter mutations (35).

## I-BET151 Is Effective Against Various Cancers

I-BET151 was first used for leukemia treatment, and later studies found that I-BET151 is also effective against various solid cancers, including breast cancer, glioma, and melanoma. Here, we summarize the anticancer activity of I-BET151 against various cancers (**Table 1**).

**Table 1:** Anticancer activity of I-BET151 against various cancer cells.

Cancer type	Cell lines	Molecular target	Effect	References
Acute myeloid leukemia	*In vitro* in MV4;11, MOLM13 and NOMO1 cell lines. *In vivo* in mice.	Inhibit *BCL2, C-MYC* and *CDK6*.	Cause G0/G1 arrest and induce cell apoptosis	(7)
Acute myeloid leukemia	*In vitro* SEM, RS4; 11 and ALL-PO cell lines. *In vivo* in mice.	Inhibit HOXA7/9 and RUNX1.	Inhibit proliferation, cause G0/G1 arrest, block cell division and induce cell apoptosis.	(36)
Acute myeloid leukemia	*In vitro* in MOLM13 and THP1 cell lines. *In vivo* in humanized bone marrow xenograft model of secondary MLL-AF9-driven B-ALL.	Inhibit Bax, BCL2 and C-MYC. Upregulate CDKN1A and CDKN1B.	Inhibit proliferation, cause G0/G1 arrest and induce cell apoptosis.	(37)
Acute myeloid leukemia	*In vitro* in OCI-AML3, KG-1, SKM1, Kasumi, ME-1 cell lines. *In vivo* in mice.	Inhibit BCL2, C-MYC and IRF8.	Cause cell cycle arrest and induce cell apoptosis.	(38)
Acute myeloid leukemia	*In vitro* in AML lines with dual DNMT3A^R882H^ and RAS mutations. *In vivo* in mice.	Inhibit Mn1, Mycn and Bcl2.	Cause cell cycle arrest and induce cell apoptosis.	(39)
Acute myeloid leukemia	*In vitro* in U937, HL-60, R-U937 and R-HL-60 cell lines.	Inhibit HP1γ.	Inhibit proliferation.	(40)
Myeloma	*In vitro* in H929, KMS12PE, KMS12BM, KMS18, KMS11 and RPMI8226 cell lines. *In vivo* in mice.	Downregulate *MYC*. Upregulate *HEXIM1*.	Cause cell cycle arrest and induce cell apoptosis.	(41)
Myeloma	*In vitro* in U266, RPMI8226, MM1 and KMS11 cell lines.	Inhibit *MYCL1* in U266. Inhibit *c-MYC* in RPMI8226, MM1 and KMS11.	Inhibit proliferation and cause cell cycle arrest.	(42)
Myeloma	*In vitro* in SKO-007(J3), CD138+ multiple myeloma cells and NK cells isolated from the bone marrow of multiple myeloma patients.	Inhibit *IRF4* and upregulate MICA.	Promote NK cell degranulation.	(29)
Myeloma	*In vitro* in RAW 264.7 cell lines and in mononuclear cells isolated from healthy donors and patients with multiple myeloma.	Inhibit *TRACP, MMP9, Ctsk and c-Src*. Upregulate OPG. Suppress IκB-α degradation and p65 nuclear translocation.	Inhibit osteoclast formation and inflammatory cytokine secretion.	(11)
Primary effusion lymphoma	*In vitro* in BC1, BC3 and BCBL1 cell lines. *In vivo* in mice.	Inhibit c-Myc.	Inhibit proliferation and cause G0/G1 arrest.	(43)
Mantle cell lymphoma	*In vitro* in JVM-2, MINO, Z138 and KPUM-YY1 cell lines	Inhibit *PAX5, IKZF1, BTK, SYK, EBF1* and *MYC*.	Cause G1/S arrest and induce cell apoptosis.	(44)
Myeloproliferative neoplasms	*In vitro* in a human erythroleukemic cell line.	Inhibit *LMO2*.	Inhibit proliferation and induce cell apoptosis.	(45)
Triple-negative breast cancer	*In vitro* in MDA-MB-231, MDA-MB-468 and BT549 cell lines.	Inhibit IKBKE.	Inhibit proliferation and induce cell apoptosis.	(12)
Breast cancer	*In vitro* in MB-231, MB-468 and SK-BR-3 cell lines.	Upregulate GSSG and MDA levels.	Induce ferroptosis.	(46)
Breast cancer	*In vivo* in mice implanted with Mvt1 and 6DT1.		Inhibit proliferation.	(47)
Glioma	*In vitro* in U87MG, A172, SW1783 cell lines and glioblastoma stem cells derived from patients. *In vivo* in mice.		Inhibit proliferation and cause G1/S arrest.	(48)
Glioma	*In vitro* in U87MG, A172, LN18, T98G cell lines and in patient derived xenograft cells.	Inhibit HOTAIR, TUG1 and H19.	Inhibit proliferation.	(49)
Glioma	*In vitro* in U87MG, U251 cell lines and Primary cells obtained from GBM patients. *In vivo* in mice.	Inhibit Notch1/NICD/Hes1.	Reduce self‐renewal and proliferation of glioma-initiating cells.	(6)
Melanoma	*In vitro* in Me1007, SK-Mel-28, Mel-RMu, Mel-JD, Mel-RM and the resistant (post) cell lines from patients. *In vivo* in mice.	Inhibit p50, p105 and CDK6. Upregulate of CDKN1A.	Inhibit cytokine/chemokine production, cause cell cycle arrest and induce cell apoptosis.	(4)
Melanoma	*In vitro* in Mel-RMU, Sk-Mel-28, Mel-RM, Mel-JD and Me1007 cell lines. *In vivo* in mice.	Inhibit XIAP. Upregulate of BIM and p21.	Cause cell cycle arrest and induce cell apoptosis.	(50)
Neuroblastoma	*In vitro* in SK-N-BE (2) and Kelly cell lines.	Inhibit *NCYM* and *N-Myc*. Upregulate of TP53INP1.	Induce cell apoptosis.	(51, 52)
Ovarian cancer	*In vitro* in 28 ovarian cancer cell lines. *In vivo* in mice.	Inhibit FoxM1, AURKB, survivin, cyclinB and PLK1.	Inhibit proliferation, cause G0/G1 arrest.	(5)
Ovarian cancer	*In vitro* in SK-OV-3, CaoV-3 and ID8 cell lines. *In vivo* in mice.	Upregulate BIM and Cleaved caspase-3. Inhibit MMP2, MMP9 and p-Stat3.	Inhibit proliferation, invasion and migration, induce cell apoptosis and antitumor immune response.	(30)
Ovarian cancer	*In vitro* in A2780CP, OVCAR3 and SKOV3 cell lines.	Inhibit FoxM1, AURKB, cyclinB1, ZEB2, N-cadherin, Survivin and Bcl-2.	Inhibit proliferation, invasion, migration and induce cell apoptosis.	(53)
Colorectal cancer	*In vitro* in HCT116 transduced with SLUG or SNAIL retroviruses.		Suppress EMT and inhibit SP cell production.	(54)
Castration-resistant prostate cancer	*In vitro* in LNCaP95 and VCaP cell lines.	Inhibit AR-V7, C-MYC, PSA and TMPRSS2.	Inhibit proliferation and AR signaling.	(55)
Pancreatic ductal adenocarcinoma	*In vitro* in human primary pancreatic stellate cells.	Inhibit COL1A1, COL1A2 and collagen I.	Attenuate fibrosis.	(56)
Non-small cell lung carcinoma	*In vitro* in Calu-1, H460 and A549 cell lines.	Inhibit c-Myc, eIF4E and cyclin D1.	Inhibit proliferation.	(57)

### Hematological Malignancies

I-BET151 exerts anti-leukemia activity by decreasing the presence of BRD4 and CDK8 in the enhancer region and downregulating the genes related to super-enhancers (SEs) (58). Although I-BET151 treatment simultaneously dissociates BRD2, BRD3, and BRD4 from chromatin, BRD4 is the most susceptible BET protein. Specifically, in I-BET151-susceptible cell lines, the inhibitor mainly affects BRD4 and prolongs the suspension of RNA Pol II (59).

A special type of acute leukemia is caused by the translocation of the *MLL* gene encoding an MLL fusion protein, which can transform hematopoietic cells into leukemia stem cells, typically resulting in poor prognosis (60). I-BET151 inhibits the transcription of *BCL2*, *C-MYC*, and *CDK6* by interfering with the chromatin recruitment of BRD3/4, which ensures efficacy in different MLL fusion cell lines and impairs the propagation of leukemia stem cells (7, 61). Administration of I-BET151 at 30 mg/kg in mouse models of MLL-AF9^+^ and MLL-AF4^+^ leukemia delays disease progression and significantly prolongs survival (7). Acute lymphocytic leukemia (ALL) in infants with MLL rearrangement is very invasive. In the preclinical mouse model of MLL-AF4^+^ infant acute lymphoblastic leukemia, I-BET151 downregulates the transcription of the *BRD4, HOXA7/HOXA9*, and *RUNX1* gene network, which reduces the disease burden. In addition, I-BET151 increased the susceptibility of MLL-rearranged ALL cells to prednisolone *in vitro*, which provides a new treatment strategy for glucocorticoid-resistant ALL (36). In the MLL-AF9^+^ cell line, the *HOXA* gene is not downregulated by I-BET151. Comparative analysis of ChIP-seq data and RNA-seq data indicates that I-BET151 only targets less than 1/10 of MLL-AF9 directly targeted genes. Treatment with I-BET151 significantly delayed the progression of lymphocytic leukemia in NSG mice (37). The mice were implanted with ceramic scaffolds of human mesenchymal stem cells, which fully simulated the environment for human bone marrow, but it was not enough to completely eradicate leukemia cells (62). This suggests that the human bone marrow-like environment may have protective properties for leukemia cells.

I-BET151 is effective against a variety of acute myeloid leukemia (AML) subtypes (38). NPM1c AML is one of the most frequently reported subtypes, and its prognosis is related to synergistic mutations (63). However, regardless of the nature of the cooperative mutation, *in vitro* and *in vivo* analyses indicate that NPM1c AML is consistently susceptible to I-BET151 because the drug inhibits BRD4 rather than wild-type NPM1 (38). Somatic mutations in DNA methyltransferase 3A (DNMT3A^mut^) occur in a variety of hematological malignancies, including in AML and elderly individuals with clonal hematopoiesis, with hot-spot mutations at the Arg882 residue (DNMT3A^R882mut^) accounting for 50–60% among the identified DNMT3A^mut^ in AML (64–67). I-BET151 causes the downregulation of DNMT3A^R882H^-related target genes by blocking BRD4; it also induces the upregulation of apoptosis-related genes and the downregulation of cell cycle progression genes. I-BET151 significantly delays the development of AML phenotypes, such as splenomegaly, increases the white blood cell count, and decreases the red blood cell count in an AML mouse model induced by two mutations, DNMT3A ^R882H^ and RAS ^G12D^ (39). The DNA methyltransferase inhibitor 5-azacytidine (AZA) is effective in myelodysplastic syndromes and AML (68). HP1γ is important in the survival of AZA drug-resistant cells, and I-BET151 can function as HP1γ inhibitor for the treatment of AZA drug-resistant hematological malignancies (40).

Critical mechanisms employed by BET inhibitors to fight multiple myeloma involve the inhibition of *MYC* transcription and MYC carcinogenesis (69), both of which are also caused by I-BET151 that exerts its inhibitory activity by attenuating the chromatin recruitment of CDK9 in a BRD2/3/4-dependent manner, which caused transcription inhibition of MYC and MYC carcinogenic programs. However, I-BET151 upregulates *HEXIM1* transcription, which leads to cycle arrest and apoptosis of myeloma cells (41). The *C-MY*C- expressing myeloma cell lines are inhibited by I-BET51, which exerts its inhibitory activity by diminishing the *c-MYC* expression, but in U266 cells that do not express *c-MYC*, I-BET151 interferes with *MYCL* expression (42, 70). I-BET151 can also target the RANKL-NF-κB signaling pathway, inhibit the formation of osteoclasts, reduce the levels of osteoclast-specific genes *TRACP, MMP9, Ctsk, and c-Src*, and inhibit the secretion of inflammatory cytokines (11).

Primary exudative lymphoma (PEL) is an aggressive non-Hodgkin’s lymphoma, which is related to Kaposi’s sarcoma-associated herpesvirus (KSHV) infection. Non-PEL cell lines are much less susceptible to I-BET151 treatment than PEL cell lines, in which the drug downregulates the c-MYC level, inhibits lymphoma cell proliferation, and induces cell cycle arrest (43). Mantle cell lymphoma (MCL) is a refractory B-cell lymphoma caused by the translocation t (11, 14)(q13; Q32) (71). BRD4 directly regulates a series of genes related to the B cell receptor signaling pathway. I-BET151 promotes the G1/S cell cycle arrest and apoptosis in BRD4-induced MCL cells, which represents a new strategy for treating MCL disease (44).

The human erythroid leukemia (HEL) cell lines are susceptible to I-BET151, which functions as a JAK2 inhibitor and remains effective against JAK2 inhibitor-resistant HEL cells (45).

### Breast Cancer

TNBC is the most aggressive breast cancer subtype, but I-BET151 can diminish NF-κB signaling by reducing *IKBKE* expression, which has a therapeutic effect on TNBC (12). High SIRT1 activity promotes DNA repair and cell cycle arrest and prevents various stress-induced apoptosis (72). I-BET151 increases the level of SIRT1 in MCF-7 and MDA-MB-231 cells, but it does not affect or even reduces the relative deacetylation activity of SIRT1 in the cells (73). I-BET151 is also known to induce ferroptosis in breast cancer cells (46). In mice implanted with highly metastatic breast cancer cell lines Mvt1 and 6DT1, I-BET151 inhibited the growth of primary tumors, but not the metastasis, which is related to the opposite effects of two BRD4 isoforms (47). Specifically, metastasis is diminished by the long BRD4 isoform but promoted by the short BRD4 isoform (74, 75).

### Glioma

Gliomas have significantly higher BRD2 and BRD4 levels than control tissues, and the mRNA and expression levels of BRD4 are closely related to the tumor subtypes and the overall survival rate of the patients, indicating that I-BET151 can have a therapeutic effect on gliomas (6, 48). I-BET151 is known to inhibit the proliferation of U87MG cells, limit the cell cycle progression from G1 to S, and reduce the tumor size in U87MG xenografts (48). There is growing evidence that long non-coding RNA plays an important role in carcinogenesis and anticancer pathways (76–78). HOX transcribed antisense RNA (HOTAIR) is overexpressed in glioma and associated with the proliferation and periodic progression of this tumor. The anticancer effect of I-BET151 in glioma is achieved, at least in part, by downregulating HOTAIR (49). Notch signaling is involved in the self-renewal of glioma stem cells (GSCs) and the regulation of tumorigenesis. The direct association between BRD4 and the Notch1 promoter region contributes to transcriptional regulation. Therefore, I-BET151 can regulate the Notch signal transduction pathway by targeting BRD4, which affects the self-renewal of GSCs and tumorigenesis (6).

### Melanoma

NF-kB is activated in melanoma (79). I-BET151 inhibits NF-kB activation in melanoma by targeting BRD2, causing cycle arrest, promoting apoptosis, and inhibiting the production of cytokines (e.g., IL6 and IL-8) and chemokines (e.g., CXCL10 and CCL5), which indicates that I-BET151 may have a therapeutic effect on melanoma (4). Another report shows that I-BET151 activates the BIM protein, a BH3-only pro-apoptotic protein family member, and the increase in BIM mediates caspase-dependent apoptosis, which is mainly related to the inhibition of BRD2. However, I-BET151-induced G1 arrest is associated with BRD4 inhibition and mediated by p21. The efficacy of I-BET151 is not identical across different melanoma cell lines; the NRAS mutant cell line (Mel-RM) and the NRAS/BRAF wild-type (Me1007) line are the most susceptible cell lines, whereas the NRAS mutant/BRAF wild-type (Mel-JD) line and the NRAS wild-type/BRAF mutant cell lines (SK-Mel-28, Mel-RMU) are relatively insensitive (50).

### Neuroblastoma

Neuroblastoma is the most common extracranial solid tumor in children, accounting for 15% of the total tumor deaths in children (80). Statistical analysis of neuroblastoma specimens shows that low expression of nuclear protein 1 induced by tumor protein 53 (TP53INP1) in tumor tissues and high expression of N-Myc in neuroblastoma patients are closely related to poor prognosis. I-BET151 inhibits the transcription and expression of *NCYM* and *N-Myc* in neuroblastoma cells and significantly increases the mRNA and protein levels of TP53INP1, which promotes apoptosis of tumor cells (51, 52).

### Ovarian Cancer

The expression of BRD4 is significantly higher in clinical ovarian cancer tissues than in non-malignant control tissues, whereas the levels of BRD2 and BRD3 do not significantly vary between malignant and non-malignant tissues. In addition, a pan-cancer analysis indicates that ovarian cancer is the most apparent tumor with BRD4 amplification. I-BET151 inhibits the viability of a wide range of ovarian tumor cells, including 28 epithelial ovarian cancer (EOC) cell lines that cover all histological types. This broad spectrum of activity is related to I-BET151-induced apoptosis mediated by mitochondria and the downregulation of the transcription and translation of FoxM1 and its transcription targets (5, 30). In addition, I-BET151 reduces the migration and invasion of EOC cells by inhibiting the Stat3 signaling pathway and downregulating ZEB2 and N-cadherin, which also inhibits tumor metastasis in the abdominal metastasis model of ovarian cancer (30, 53). Similar to the discovery in multiple myeloma, I-BET151 can also induce anticancer immunity in ovarian cancer (30).

### Colorectal Cancer

Mutation or defect of succinate dehydrogenase B (SDHB) can lead to the loss of enzyme activity and expression, which can occur in various malignant tumors. However, colorectal cancer cells with SDHB knockout are highly susceptible to I-BET151 (81). EMT cells were generated from colorectal cancer tissue by SLUG or SNAIL retrovirus transduction, which also produced side population (SP) cells with low Hoechst 33342 staining and differentiation potential. However, I-BET151 not only inhibits the occurrence of EMT but also reduces the proportion of produced SP cells (54).

### Prostatic Cancer

Androgen receptor (AR) is the main carcinogen in the development of prostate cancer. Second-generation antiandrogen therapy can enhance receptor signaling and improve the prognosis of castration-resistant prostate cancer (CRPC) (82–84). However, the expression of AR splice variants leads to drug resistance, including the AR splice variant 7 (AR-V7) (85, 86). Based on clinical prostate samples, BRD4 is associated with AR activity and patient survival. I-BET151 decreases AR-V7 and C-MYC expression levels and inhibits AR signaling, suggesting a new therapeutic strategy for patients with CRPC (55).

### Pancreatic Ductal Adenocarcinoma

Pancreatic ductal adenocarcinoma (PDAC), as the most common pancreatic cancer type, is often associated with the development of a fibrotic reaction (87). Pancreatic stellate cells (PSCs) are the key regulators of fibrosis that produce only limited amounts of collagen I in the static state (88). However, unlike BRD2, BRD3, and the FOS-like 1 (FOSL1) protein, BRD4 promotes collagen I production in primary prostate cancer isolated from human PDAC. I-BET151 can effectively suppress the fibrotic reaction and collagen I production by inhibiting BRD4 and preventing BRD4-mediated blockage of FOSL1 (56).

### Non-Small Cell Lung Carcinoma

Eukaryotic translation initiation factor 4E (eIF4E), a component of the translation initiation complex, is associated with cellular survival, EMT, and angiogenesis (89, 90). I-BET151 inhibits BRD4 and, therefore, downregulates eIF4E, causing dose-dependent inhibition of cell growth in non-small cell lung cancer (57).

## Anticancer Efficacy of I-BET151 in Combination With Other Drugs

Drug resistance or insensitivity is a critical clinical issue associated with chemotherapy in cancer treatment. To overcome drug resistance and improve anticancer efficacy, an increasing number of experiments have been conducted to test I-BET151 in combination with other drugs (**Table 2**).

**Table 2:** Combination of I-BET151 and other drugs in cancer.

Types of Combination	Cell lines	Molecular target	Effect	References
I-BET151 and trametinib	*In vitro* in SUM-159PT and MDA-MB-231 cell lines. *In vivo* in mice.	Inhibit trametinib-induced PDGFRB and DDR1 (SUM-159PT) and FGFR2 and DDR1 (SUM-229PE).	Inhibit trametinib-induced growth and prevent or reverse adaptive drug resistance of cancer cells to trametinib.	(91)
I-BET151 and TMZ	*In vitro* in U87MG and U251 cell lines. *In vivo* in mice.	Upregulate PUMA.	Promote TMZ-induced apoptosis, oxidative stress and suppress migration, invasion, and formation of colony.	(92)
I-BET151 and S63845	*In vitro* in 11 melanoma cell lines such as A06M, C002M, C025M1, etc. *In vivo* in mice.	Inhibit BCL2A1, upregulate BIM and induce caspase‐dependent death.	Synergistically induce apoptosis and expansion of the range of action of I-BET151 and S63845.	(93)
I-BET151 and alisertib	*In vitro* in NB-1643, SK-N-SH, NB-SD and SK-N-AS cell lines. *In vivo* in mice.	Inhibit reflexive upregulation of AURKA, MYC and MYCN in response to alisertib.	Synergistically inhibit neuroblastoma viability *in vitro* and vivo.	(94)
I-BET151 and IKK inhibitor VII	*In vitro* in U937 and I-BET151-resistant U937 cell lines.	Inhibit NF-κBp65.	Enhance or restore the sensitivity to I-BET151 in U937 cells.	(13)
I-BET151 and THZ1	*In vitro* in K562, Jurkat and murine I-BET151-resistant AF9 AML cells.	Synergistically inhibit of the re-activated MYC, MYB, TAL1 and LMO2.	Synergistically induce anticancer effect toward I-BET151-resistant leukemia.	(95)
I-BET151 and Vitamin C	*In vitro* in MDA-MB-231, BT-549 and HCC1937 cell lines.	Upregulate HDAC1 and inhibit H3ac and H4ac.	Sensitize TNBC to I-BET151.	(96)
I-BET151 and Vitamin C	*In vitro* in 1205Lu, C8161, SK-MEL-28, A2058 and SK-MEL-2 cell lines.	Inhibit HAT1 and the acetylation of H4.	Sensitize melanoma to I-BET151.	(97)
I-BET151 and LBH589	*In vitro* in KMJR138, Me1007, Mel-RM cell lines and cells from patients. *In vivo* in mice.	Inhibit the AKT and Hippo/YAP signaling pathways. Upregulate BIM.	Synergistically induce caspase-dependent apoptosis.	(98)
I-BET151 and LBH589	*In vivo* in mice.		Upregulate antileukemic activity.	(36)
I-BET151 and romidepsin	*In vivo* in mice.	Increase IL-6 production and enhance CD8+ T cell proliferation.	Upregulate vaccine-elicited Ab responses.	(99)
I-BET151, Forskolin, ISX9, CHIR99021 and DAPT	*In vitro* in U87MG and glioblastoma stem cells.	Upregulate *Ngn2, Ascl1, Brn2* and *MAP2*.	Reprogram of glioblastoma cells into Neurons.	(100)
I-BET151, forskolin and rapamycin	*In vitro* in U87MG and C6.	Inhibit *pdgfra, pdgfrb, pdgfrl, met, vegfa and colla1*.	Suppress proliferation and reprogram malignant gliomas to differentiate into glial cells.	(101)

In TNBC patients who received trametinib for one week, an adaptive bypass reaction leading to trametinib resistance can be observed. The combination of I-BET151 and trametinib not only synergistically inhibits TNBC growth *in vitro* and *in vivo* but also prevents or reverses adaptive drug resistance of cancer cells to trametinib (91). In clinical practice, temozolomide (TMZ) is the main treatment for malignant glioma, but the drug resistance of glioma cells to TMZ will lead to treatment failure. I-BET151 can promote TMZ-mediated inhibition of glioma proliferation, invasion, and migration, enhance the oxidative stress induced by TMZ, and restore susceptibility of glioma cells to TMZ, all of which may be related to the I-BET151-induced expression of the p53-upregulated modulator of apoptosis (PUMA) (92). S63845 is a myeloid leukemia cell differentiation protein 1 (MCL1) inhibitor with therapeutic efficacy against a large number of melanoma cell lines, excluding a few that are not affected by MCL1 inhibition. The combination of this inhibitor with I-BET151 is superior to any single treatment, especially related to the caspase-dependent cell death induced by this combination (93). Neuroblastoma is mainly driven by MYC or MYCN. Inhibition of Aurora kinase A (AURKA) is an effective treatment, but treatment with an AURKA inhibitor (alisertib) often causes an upregulation of the transcription of AURKA, MYC, and MYCN. The combination of the AURKA inhibitor with I-BET151 significantly reduces the transcriptional upregulation and synergistically inhibits the tumor cell survival (94).

The mechanism of I-BET151 resistance of lymphoma cell line U937 is known to be related to the activation of NF-κB. The combination treatment with IKK inhibitor VII can inhibit the activation of NF-κBp65 protein in the nucleus of drug-resistant cells and enhance or restore the susceptibility of U937 cells to I-BET151 by targeting the NF-κB signaling pathway (13). MYC is significantly inhibited in I-BET151-susceptible cells, but it is not affected in I-BET151-resistant cells, indicating a functional compensation for MYC in I-BET151-resistant AML cells, which is related to enhancer remodeling. Plasmacytoma variant translocation 1 (PVT1) is a long non-coding RNA (lncRNA) that functions as an oncogene in many cancers and is known to promote MYC expression in I-BET151-resistant AML cells in a BRD4-independent manner (95, 102). Furthermore, CDK7 inhibitor (THZ1) inhibits MYC expression by interfering with RNA polymerase II activity of the PVT1 enhancer, which kills cancer cells in combination treatment with I-BET151 (95). Interestingly, vitamin C also improves the efficacy of I-BET151. In TNBC cells, the upregulation of histone deacetylase 1 (HDAC1) and the inhibition of histone H3 and H4 acetylation by vitamin C enhances the effect of I-BET151, as indicated by a lower half-maximal effective concentration (EC_50_), which allows a dose reduction of I-BET151 and concomitantly decreases the risk of side effects (96). However, vitamin C also diminishes the expression of histone acetyltransferase 1 (HAT1) and limits the acetylation of lysine 5 and lysine 12 on H4 without reducing the acetylation of H3 (97). These specific effects can be potentially related to the origin of the tumors from different tissue types, but they also indicate that the I-BET151 activity can be improved by mediating histone acetylation. Thus, combinations of I-BET151 and histone deacetylase (HDAC) inhibitors are increasingly used in patients. In melanoma, the combination of I-BET151 and HDAC inhibitor LBH589 effectively inhibits the AKT and Hippo/YAP signaling pathways, upregulates the BIM expression, synergistically induces caspase-dependent apoptosis of tumor cells, and significantly prolongs the survival time in a xenograft *in vivo* model (98). This combination also has a synergistic anti-leukemia effect in the preclinical mouse model of MLL-AF4+ infant ALL (36). In melanoma, the replacement of LBH589 with romidepsin for combination therapy with I-BET151 promotes apoptosis and changes the expression of IL-6/JAK/STAT-related genes, which increases the response frequency of CD8+ T cells in mice vaccinated with OVA+CpG tumor vaccine and improves the treatment efficacy and preventive protection of the vaccine (99).

Gliomas originate from glial precursor cells that can be reprogrammed to neurons with the help of nerve cell-specific transcription factors (103–105). Several small molecule combinations involving I-BET151 are known to treat malignant gliomas. The combination of cAMP enhancer Forskolin, ISX9, CHIR99021, and I-BET151, along with dual antiplatelet therapy (DAPT), can upregulate the expression levels of the *Ngn2*, *Ascl1*, *Brn2*, and *MAP2* genes in U87 MG cells and reprograms the tumor cells to neuronal morphology without undergoing the intermediate pluripotent state, which can lead to the inhibition of U87MG cell growth and the formation of tumor-like spheroids (100). Another experiment also demonstrates that the combination treatment consisting of I-BET151, along with Forskolin and mammalian target of rapamycin (mTOR) inhibitor (rapamycin), can also reprogram malignant glioma cells into non-proliferative glial cells and strongly inhibit the proliferation of tumor cells. Although this combination is only effective in some glioma types, its inhibitory effect on glioma proliferation is stronger than that of TMZ, and it can still be used in TMZ resistant cells (101).

## Conclusion

This review presents a discussion of the anticancer effects and mechanisms of I-BET151, which specifically targets BRD2 and BRD4, regulates the pathways of NF-κB, Notch, and Hh signal transduction, change TME and controls the telomere length. These I-BET151-mediated mechanisms cause the inhibition of proliferation, migration, and invasion of cancer cells, along with the induction of apoptosis. We also assessed the effects of I-BET151 used in combination with other drugs, and we describe different combination types that substantially increase the sensitivity of select chemotherapy drugs and achieve an improved therapeutic efficacy.

I-BET151 has a wide application prospect, and the most attractive one is its application in glioma. The combination of Forskolin, ISX9, CHIR99021, I-BET151 and DAPT can treat glioma by changing the differentiation state of cancer cells and reprogramming glioma cells into neurons. The process did not go through an intermediate pluripotent state, which means that the formed neurons are much less likely to become cancer cells again. This therapeutic strategy is expected to change the current treatment mode of glioma. For patients with small glioma and unobvious space-occupying effect, the use of this drug combination can promote tumor cell transformation and avoid the trauma caused by surgery. On the other hand, for patients with obvious space-occupying effects, surgical resection is required, and then the remaining tumor cells are converted into neurons by using the medicine combination, which can reduce the damage to healthy brain tissue caused by excessive surgical resection range. Moreover, compared with the emerging gene therapy, the side effects caused by drug therapy are easier to be found and solved, and the economic and technical costs required for treatment are relatively lower. This therapeutic strategy has provided new ideas for the clinical treatment of glioma and inspired the treatment of other types of cancer.

At present, there is no related clinical trials, which may be due to the short development time of I-BET151. However, I-BET151 is a valuable anticancer drug with a wide range of therapeutic effects based on preclinical experiments, which provides us with a new therapeutic strategy for clinical anticancer treatment.

## Publisher’s Note

All claims expressed in this article are solely those of the authors and do not necessarily represent those of their affiliated organizations, or those of the publisher, the editors and the reviewers. Any product that may be evaluated in this article, or claim that may be made by its manufacturer, is not guaranteed or endorsed by the publisher.

## Author Contributions

JL wrote the article. ZL and YZ collected and organized data and figures. CM and HH revised the manuscript critically. All authors contributed to the article and approved the submitted version.

## Funding

This work was supported by the Natural Sciences Foundation of Jilin Province (20180101158JC).

## Conflict of Interest

The authors declare that the research was conducted in the absence of any commercial or financial relationships that could be construed as a potential conflict of interest.

## Publisher’s Note

All claims expressed in this article are solely those of the authors and do not necessarily represent those of their affiliated organizations, or those of the publisher, the editors and the reviewers. Any product that may be evaluated in this article, or claim that may be made by its manufacturer, is not guaranteed or endorsed by the publisher.
